# Integrating generative AI into physiotherapy education: students’ use, perceptions, and generative AI literacy following curricular adaptations: a repeated cross-sectional programme evaluation

**DOI:** 10.1186/s12909-026-10256-0

**Published:** 2026-08-28

**Authors:** Karin Schröder, Yvonne Lindbäck, Torkel Engström, Sofi Sonesson

**Affiliations:** Unit of Physiotherapy, Department of Health, Medicine and Caring Sciences, Linköping University, Linköping, Sweden

**Keywords:** Generative Artificial Intelligence, Medical Education, Physical Therapy Modalities, Students, Health Occupations, Clinical Competence

## Abstract

**Background:**

Structured generative artificial intelligence (GAI) training has been proposed as an effective approach to strengthen healthcare students’ confidence in navigating AI-related challenges and ethical considerations. However, few studies have evaluated the impact of implemented GAI-related curricular adaptations and learning activities, particularly within physiotherapy education. This study aimed to evaluate: 1) physiotherapy students’ use and perceptions of GAI in their studies during implementation of GAI-related curricular adaptations; and 2) GAI literacy in semester 1 before participation and in semester 3 after completion of structured GAI learning activities.

**Methods:**

A two-part repeated cross-sectional programme evaluation with non-equivalent, unlinked samples was conducted among physiotherapy students at Linköping University, Sweden. Students’ use and perceptions of GAI were assessed after implementation of GAI-related curricular adaptations in 2024 and again in a separate survey wave in 2025. GAI literacy was evaluated using the 10-item Generative Artificial Intelligence Literacy Scale (GAILS-10) before and after the GAI learning activities delivered during semesters 1–3.

**Results:**

Reported GAI use differed significantly between 2024 and 2025 surveys, χ^2^(3) = 62.40, *p* < 0.001, with daily use reported by 57% of respondents in the 2025 survey compared with 13% in the 2024 survey. Respondents in the 2025 survey reported more diverse use of GAI, including brainstorming, question generation, and text refinement (*p* < 0.001). Perceptions of GAI as contributing to learning were also more positive in the 2025 survey, χ^2^(4) = 24.94, *p* < 0.001, whereas perceptions of information about permissible and impermissible use were similar in both surveys. Semester 3 students assessed after completing the GAI learning activities reported higher GAILS-10 scores across all dimensions than semester 1 students assessed before participation, with a large between-group effect size for the total score (*d* = 1.0).

**Conclusion:**

Physiotherapy students reported more frequent and diverse GAI use and more positive learning perceptions in the 2025 survey than in the 2024 survey. Semester 3 students, assessed after completing the GAI learning activities, reported higher GAI literacy than semester 1 students assessed before participation in these activities. These findings suggest a need for curricular adaptations and educational support to promote appropriate GAI use, reduce misuse, and strengthen student GAI literacy.

**Supplementary Information:**

The online version contains supplementary material available at https://doi.org/10.1186/s12909-026-10256-0.

## Background

Generative Artificial Intelligence (GAI) is transforming how students in healthcare education access, process, and engage with knowledge [1]. Despite its growing integration and sophistication, both educators and students often lack a comprehensive understanding of GAI’s implications, limitations, and ethical considerations [2, 3]. This gap highlights the urgent need to redesign learning activities, assessments, and curricula to support the responsible and effective use of GAI.

GAI offers several educational advantages, including rapid access to information, support in summarising and enhancing text, and personalised feedback [4]. These features have been shown to improve learning outcomes, skills development, student engagement, and academic performance [5, 6]. Moreover, GAI can provide individualised learning experiences tailored to students’ needs [7, 8]. Most healthcare students recognise the potential benefits of GAI both in their academic studies [9] and in future clinical practice [10]. However, unregulated use of GAI in medical education may undermine critical thinking, problem-solving, and in-depth knowledge, especially when students rely heavily on AI-generated content without proper guidance [11, 12]. Despite frequent use, healthcare students have expressed a need for clearer guidelines, ethical frameworks, and foundational knowledge about AI technologies [10, 13, 14].

‘GAI literacy’ refers to the skills, knowledge, and confidence required to use GAI technologies effectively and responsibly, encompassing technical operation, creative application, critical evaluation, ethical awareness, and collaborative communication [15, 16]. Structured GAI training has been suggested to enhance health care students’ confidence in navigating AI-related concepts, challenges, and ethical considerations [10, 17]. Although GAI literacy is recommended across all healthcare disciplines, few studies have assessed the effectiveness of related learning activities. While nursing and medical education have increasingly integrated and evaluated GAI-supported learning approaches [18–24], evidence within physiotherapy education remains comparatively limited and underdeveloped [25]. Emerging studies in physiotherapy education suggest that AI-supported learning may enhance knowledge acquisition, foster positive attitudes towards AI, and improve AI self-efficacy [26, 27], yet findings remain inconsistent and context-dependent, with some studies reporting limited student engagement and no clear advantage over conventional educational approaches [28]. Moreover, previous research highlights heterogeneity in how students engage with GAI, including variability in patterns of use, perceptions, and levels of AI literacy, alongside a need for structured curricular adaptations and clearer guidance to support appropriate and effective use [10, 29, 30]. To address this gap, this study aimed to: 1) evaluate physiotherapy students’ use and perceptions of GAI in their studies during the implementation of GAI-related curricular adaptations; and 2) evaluate students’ GAI literacy before and after a series of structured GAI learning activities.

## Methods

### Study design

**Fig. 1:**
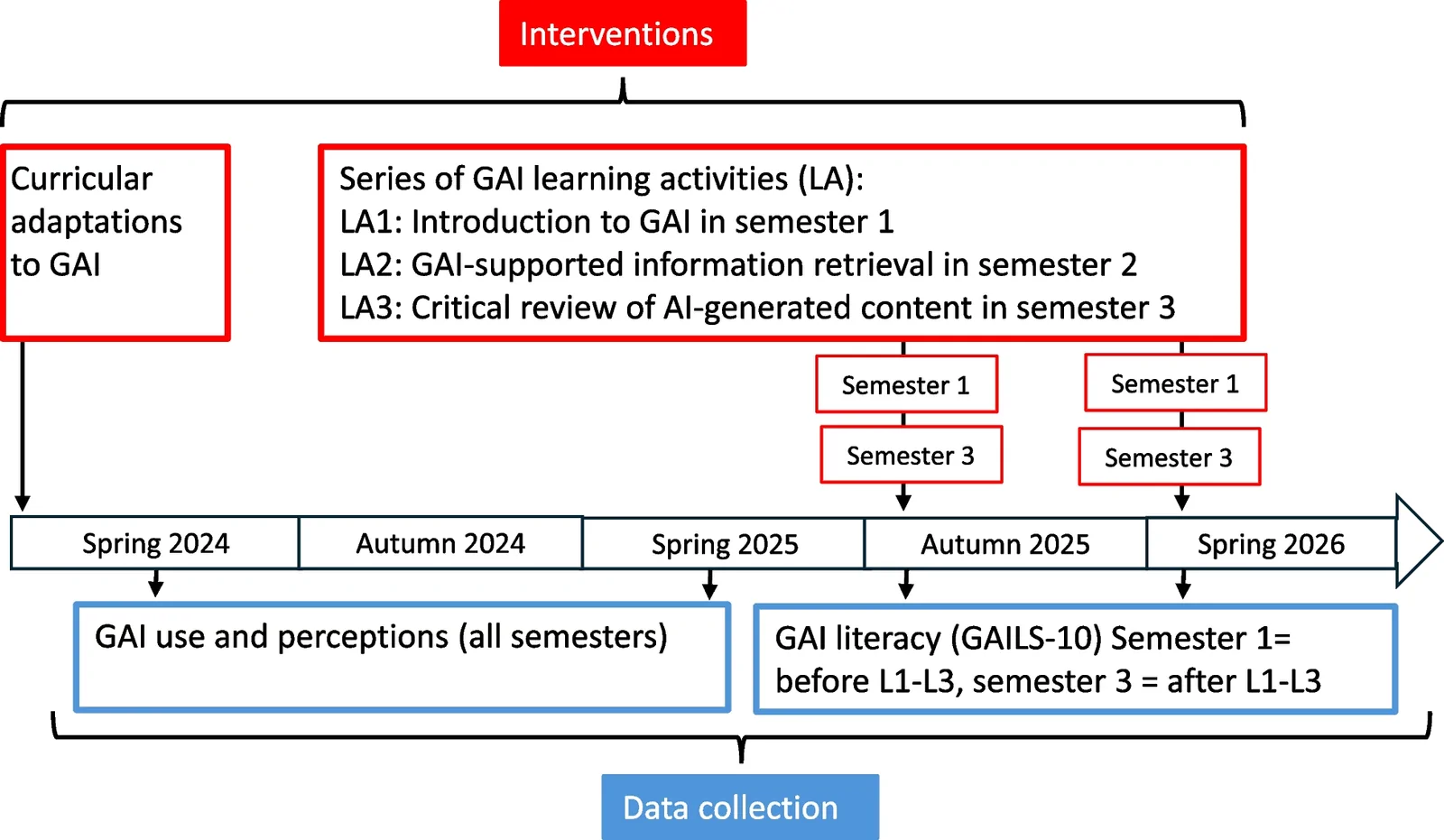
Timeline of intervention, data collection, generative artificial intelligence (GAI) use, perceptions, and literacy. GAI = Generative Artificial Intelligence, LA = Learning Activities, LA1–3 = different Generative Artificial Intelligence learning activities in semesters 1–3, GAILS-10 = 10-item Generative Artificial Intelligence Literacy Scale [32], Semesters 1–3 = different semesters in physiotherapy education

This study was designed as a two-part repeated cross-sectional programme evaluation with non-equivalent, unlinked samples of physiotherapy students. In January 2024, the physiotherapy programme implemented curricular adaptations related to GAI. A survey on students’ use and perceptions of GAI in their studies was distributed across all semesters at the time of implementation and repeated one year later. Perceptions included learning support needs, trust in AI-generated content, understanding of institutional guidance, and expectations for future use. To further enhance GAI literacy, targeted learning activities were introduced in autumn 2024 for students in semesters 1–3. These were evaluated using two cross‑sectional assessments: one administered to semester 1 students prior to any formal GAI literacy training, and a second administered to semester 3 students after completing the GAI learning activities (Fig. 1). The study followed the Guideline for Reporting Evidence‑based Practice Educational Interventions and Teaching (GREET) [31].

### Settings and participants

The setting for this GAI initiative was the Swedish physiotherapy programme at Linköping University, Sweden. The programme employs problem-based learning (PBL), a student-centred pedagogical approach in which students develop skills and knowledge by analysing authentic, real-world problems, often working in small groups [33]. PBL promotes active and self-directed learning, clinical reasoning and problem-solving skills [34]. The physiotherapy programme spans six semesters and enrols approximately 30–50 students per semester, with a larger number in the earlier semesters. In the beginning of the spring semester in 2024, a total of 224 students were enrolled, with a gender distribution that was 55% women (n = 124) and 45% men (n = 100), and a mean age of 24.8 years (SD 4.4). All students enrolled in the physiotherapy programme were eligible to participate in the GAI use and perception surveys in spring 2024 and spring 2025 and all eligible students were invited at each time point. In 2024, 224 students were enrolled and invited, of whom 123 responded (55%); in 2025, 217 students were enrolled and invited and 112 responded (51%). Because semester-specific denominators were unreliable due to variation in enrolment and student progression, semester-specific response rates are not reported; Table 1 presents the distribution of respondents across semesters. For the GAILS-10 assessment, all eligible semester 1 and semester 3 students were invited. Semester 1 students were assessed before participation in the structured GAI learning activities, and semester 3 students were assessed after completing these activities. Data were collected during autumn 2025 and spring 2026. In total, 175 of 182 eligible students participated (96%), including 95 of 98 semester 1 students (97%) and 80 of 84 semester 3 students (95%).

**Table 1:** Distribution of physiotherapy student respondents by semester in the 2024 and 2025 surveys

Semester	2024 survey, n (%)	2025 survey, n (%)
1	17 (13.8)	24 (21.4)
2	29 (23.6)	33 (29.5)
3	23 (18.7)	20 (17.9)
4	24 (19.5)	32 (28.6)
5	22 (17.9)	2 (1.8)
6	8 (6.5)	1 (0.9)
Total number in surveys	123 (100)	112 (100)

The total number of students enrolled in the physiotherapy programme was 224 students in the beginning of 2024 and 217 students in the beginning of 2025

### Intervention

#### Curriculum adaptations related to generative AI

During autumn 2023, all courses in the physiotherapy programme were reviewed using constructive alignment principles [35] and adaptations to learning and assessment tasks were introduced to reduce potential misuse of GAI. These adaptations, together with standardised text on permissible GAI use, were implemented across all courses in January 2024 (Fig. 1). The process continued throughout 2024 with the introduction and testing of GAI-adapted learning and assessment tasks, and emphasised institutional GAI guidelines in various course contexts. Further details are available in a previous publication [30].

#### Generative AI learning activities

This study implemented a structured, multi-session educational intervention to develop GAI literacy among all students in the physiotherapy programme. Integrated into existing course activities, it combined pre-recorded lectures, in-person workshops, individual learning tasks, and group-based seminars delivered across semesters 1–3 (Supplementary file 1). The curricular adaptation was designed to support GAI literacy and align with the intended learning outcomes. The learning activities gave students structured opportunities to use GAI in authentic tasks, critically appraise AI-generated outputs, consider ethical and responsible use, and reflect on its relevance for physiotherapy education and future practice. This approach supported both practical experience with GAI and critical judgement regarding its limitations, risks, and educational value. The educational intervention was based on the Ability–Motivation–Opportunity (AMO) framework [36], which provides a theoretical structure for understanding how educational interventions can facilitate the learning process and support the development of GAI literacy. According to the AMO framework, optimal work performance is achieved when three conditions are met: *Ability* (skills, knowledge and competence), *Motivation* (willingness to engage), and *Opportunity* (supportive conditions) [37]. In this study, the AMO framework served to clarify how each activity contributes to strengthening students’ GAI literacy across the three dimensions. While the AMO framework guided the design and structuring of the intervention, the learning activities were aligned with the PBL approach of the physiotherapy programme, emphasising active, student-centred learning and collaborative engagement with authentic tasks. The teaching team comprised one librarian and an experienced physiotherapy teacher who also conducts research on the integration of GAI in education.

### Outcomes

#### Generative AI use and perceptions

The survey examined students’ use and perceptions of GAI tools in their studies as well as the clarity of institutional guidelines. The questionnaire included closed-ended items on students’ use and perceptions of GAI, together with open-ended items allowing respondents to describe additional uses of GAI and suggest learning support activities. The questionnaire was developed by the authors and refined through review and discussion by researchers and educators with expertise in physiotherapy education, digital learning and GAI. Details of the questionnaire development process, including the purpose of the questionnaire, item generation, expert review, pilot-testing, content validity, measurement structure, and the full translated questionnaire with open-ended items clearly identified, are provided in Supplementary file 2. No formal pilot-testing or psychometric validation was performed prior to use. The 2024 questionnaire included 13 items, all of which were repeated in the 2025 questionnaire, with one additional question added: “To what extent do you trust the answers you receive from GAI such as ChatGPT/Copilot?” One question addressed students’ current semester. Six questions addressed students’ use of GAI, rated on a Likert-type scale with four to five response options, including one open-ended question on additional ways of using GAI. Seven questions addressed students’ perceptions of GAI. Six items were rated on a Likert-type scale (with three to six response options) and addressed the perceived usefulness of GAI for learning, need for learning support, trust in AI-generated responses, clarity of institutional guidance, engagement with the study guide, and expectations regarding future use. One open-ended question invited students to suggest GAI-related learning activities. Responses to the GAI use and perception questionnaire were analysed at the item level.

#### Generative AI literacy

GAI literacy was assessed using the Generative Artificial Intelligence Literacy (GAIL) Scale, which encompasses five dimensions: basic operational skills, prompt engineering ability, quality evaluation ability, innovative application ability, and ethical and regulatory awareness [32]. The scale is based on the AMO framework to be able to analyse how GAI literacy may influence job performance [36]. The GAIL scale has demonstrated robust psychometric validity through exploratory and confirmatory factor analysis and hypothesis testing [32]. We used the GAILS-10, a short form derived from these prior factor analyses, with two items representing each of the five domains [32]. The GAILS-10 was translated into Swedish for this study by TE, a physiotherapy educator with experience in GAI, pedagogy, and digital learning, with Microsoft Copilot used as supplementary translation support. The translated version was reviewed by the research group but was not culturally adapted or pilot-tested. Items were rated on a 7-point Likert scale (1 = strongly disagree, 7 = strongly agree), with higher scores indicating higher GAI literacy. A total score was calculated by summing item responses, yielding a range of 10 to 70.

### Data collection

The GAI use and perceptions survey was distributed as anonymous online questionnaires to all students in the physiotherapy programme during scheduled lectures in spring 2024 and spring 2025. Students completed the questionnaire on their mobile devices, and one reminder was provided at each survey wave. Participation was voluntary, and informed consent was obtained before data collection. The GAILS-10 was administered anonymously on paper during mandatory GAI learning activities. Semester 1 students completed it in week 2 before any GAI-related teaching, whereas semester 3 students completed it after having participated in the structured GAI learning activities delivered during previous semesters (Fig. 1).

### Data analysis

Data were analysed using SPSS statistical software version 29.0. A significance level of *p* < 0.05 was applied. As this was an exploratory programme evaluation, no adjustment for multiple comparisons was performed. Accordingly, findings should be interpreted as hypothesis-generating and considered alongside effect estimates and confidence intervals where applicable. Closed-ended analyses of GAI use and perceptions included all submitted questionnaires at each survey wave (*n* = 123 in 2024 and *n* = 112 in 2025), with percentages calculated using the number of submitted questionnaires as the denominator. These items were mandatory and therefore had no item-level missing data. Open-ended analyses included only students who provided written responses. Free-text responses were analysed using quantitative content analysis, whereby responses were coded into descriptive categories and category frequencies were calculated following the principles outlined by Krippendorff [38]. The coding was inductive, with categories developed from the response content rather than from a predetermined coding framework. One researcher, KS, initially reviewed and grouped responses according to similar content. Preliminary categories were then discussed within the research group (KS, YL, TE, SS) and refined through consensus to ensure clarity and alignment with the response content. As students could provide more than one response, frequencies represent coded responses rather than individual respondents. GAILS-10 analyses included all students who completed the scale (*n* = 95 in semester 1 and *n* = 80 in semester 3). Because survey responses were anonymous and unlinked, analyses were conducted as between-sample comparisons rather than paired or repeated-measures analyses. Differences between cohorts were examined with independent t-tests and Mann–Whitney U tests. Pearson’s chi-square test was used unless assumptions were violated. Fisher’s Exact Test was applied when any expected cell count was < 1 or when more than 20% of expected cell counts were < 5. Cramér’s V was reported as a measure of effect size. Interpretation of small (V = 0.10), medium (V = 0.30), and large (V = 0.50) effects followed Cohen’s benchmark thresholds for contingency tables [39].

## Results

In the GAI use and perception survey, 123 of 224 physiotherapy students (55%) responded in 2024, and 112 of 217 students responded in 2025 (51%). Responses were obtained from students across all six semesters of the physiotherapy programme. The distribution of respondents by semester is shown in Table 1. In the GAI literacy assessment (GAILS-10), 175 of 182 students (96%) participated, including 95 of 98 students (97%) in semester 1, assessed prior to participation in GAI-related learning activities; and 80 of 84 students (95%) in semester 3, assessed after completion of the learning activities.

### Generative AI use and perceptions

Reported frequency of GAI use differed significantly between the 2024 and 2025 survey cohorts, with more frequent use reported in the 2025 survey, χ^2^ (3) = 62.40, *p* < 0.001. Regular use predominated in the 2025 survey, with daily use reported by 57% of respondents compared with 13% in the 2024 survey, corresponding to a large between-survey effect size (Cramér’s V = 0.52). Reported uses of GAI were more diversified in the 2025 survey than in the 2024 survey. Respondents in the 2025 survey reported significantly greater use of GAI for brainstorming, question generation, and text refinement (*p* < 0.001), with small to large between-survey effect sizes (Cramér’s V = 0.28–0.52). However, generating new assignment text remained uncommon in both surveys, with no significant between-survey difference (Table 2). Students also reported a range of additional uses of GAI beyond the predefined categories. The most frequently reported use in both surveys was supporting understanding, reported by nearly half of respondents at both time points. Compared with the 2024 survey, the 2025 survey showed more frequent reported use of GAI for language support, creating case scenarios, and studying for exams, while non-use was less common (Table 3).

**Table 2:** Students’ use of generative artificial intelligence (GAI) in the 2024 and 2025 surveys

	**2024 survey** **(*n* = 123)**	**2025 survey** **(*n* = 112)**	**χ**^**2**^** or Fisher’s Exact Test, *p*-value**	**Cramer’s V**
	**N (%)**	**N (%)**		
How often do you use GAI such as ChatGPT/Copilot in your studies?				
Every day Once a week Once a month Never	16 (13.0) 51 (41.5) 26 (21.1) 30 (24.4)	64 (57.1) 38 (33.9) 5 (4.5) 5 (4.5)	χ^2^ (3) = 62.40 *p* < 0.001	0.52
To what extent do you use GAI such as ChatGPT/Copilot to brainstorm ideas when studying?				
Often Quite often Occasionally Rarely Never	7 (5.7) 24 (19.5) 30 (24.4) 27 (22.0) 35 (28.5)	31 (27.7) 38 (33.9) 29 (25.9) 4 (3.6) 10 (8.9)	χ^2^ (4) = 48.88 *p* < 0.001	0.46
To what extent do you use GAI such as ChatGPT/Copilot to write questions for yourself?				
Often Quite often Occasionally Rarely Never	3 (2.4) 3 (2.4) 17 (13.8) 19 (15.5) 81 (65.9)	4 (3.6) 18 (16.1) 21 (18.8) 40 (35.7) 29 (25.9)	χ^2^ (4) = 42.91 *p* < 0.001	0.43
To what extent do you use GAI such as ChatGPT/Copilot to rewrite/improve your written text to be submitted in an assignment?				
Often Quite often Occasionally Rarely Never	5 (4.1) 10 (8.1) 18 (14.6) 15 (12.2) 75 (61.0)	5 (4.5) 15 (13.4) 32 (28.6) 23 (20.5) 37 (33.0)	χ^2^ (4) = 19.02 *p* < 0.001	0.28
To what extent do you use GAI such as ChatGPT/Copilot to generate new text to be submitted as an assignment in your course?				
Often Quite often Occasionally Rarely Never	5 (4.1) 3 (2.4) 6 (4.9) 15 (12.2) 94 (76.4)	0 (–) 2 (1.8) 10 (8.9) 16 (14.3) 84 (75.0)	Fisher’s Exact = 6.23 *p* = 0.183	0.16

**Table 3:** Respondents’ additional reported uses of generative artificial intelligence (GAI)

Open-ended question” Are there any other ways you use GAI tools such as Copilot or ChatGPT in your studies, besides the previously mentioned purposes (writing questions to yourself, improving written text, generating new texts)?” *			
**Category**	**Category description**	**Frequency** **2024 survey**	**Frequency** **2025 survey**
Supporting in understanding	Explain, understand, clarify, simplify	29	28
Linguistically	Formulate, improve text, summarise, write references, find synonyms, translate	20	28
Making case scenarios and exam preparation	Study for exams, writing questions, study friend, make practice exams/quiz, create patient cases	5	10
Searching literature	Search for books and journals	2	4
Non-use	Don’t use GAI, used once	3	1
Total number of codes		59	71
*Frequencies represent coded responses; multiple responses were permitted. The question was answered by 57 students in the 2024 survey and 58 students in the 2025 survey			

Students’ perceptions of GAI differed between the 2024 and 2025 surveys. Compared with the 2024 survey, a higher proportion of students in the 2025 survey reported that GAI contributed to their learning (χ^2^ (4) = 24.94, *p* < 0.001), corresponding to a large between-survey effect size (Cramér’s V = 0.33). Most students in both surveys reported no need for additional learning support, with no significant between-survey difference. Trust in GAI was moderate in the 2025 survey, with 62.7% selecting “neither”. Perceptions of institutional guidance and clarity regarding permissible GAI use were similar in both surveys. A higher proportion of students reported having read the study guide in the 2025 survey (*p* = 0.039; V = 0.15, small effect). Expectations regarding future use were positive, with no significant differences between surveys (Table 4).

**Table 4:** Students’ perceptions of generative artificial intelligence (GAI) in the 2024 and 2025 surveys

	**2024 survey** **(*n* = 123)**	**2025 survey (*n* = 112)**	**χ**^**2**^** or Fisher’s Exact Test, *p*-value**	**Cramer’s V**
	**N (%)**	**N (%)**		
To what extent do you think GAI such as ChatGPT/Copilot has contributed to your learning?				
Very high degree High degree Neither nor Low degree Not at all	8 (6.5) 44 (35.8) 31 (25.2) 15 (12.2) 25 (20.3)	24 (21.4) 46 (41.1) 30 (26.8) 8 (7.1) 4 (3.6)	χ^2^ (4) = 24.94 *p* < 0.001	0.33
Do you need a learning support element to be able to use GAI such as ChatGPT/Copilot optimally in your learning?				
Yes Uncertain No	21 (17.1) 51 (41.5) 51 (41.5)	14 (12.5) 39 (34.8) 59 (52.7)	χ^2^ (2) = 3.07 *p* = 0.215	0.11
To what extent do you trust answers from GAI such as ChatGPT/Copilot? (Note: asked only in the 2025 survey)				
Very high degree High degree Neither nor Low degree Not at all Do not know		0 (–) 24 (21.8) 69 (62.7) 15 (13.5) 2 (1.8) 2 (1.8)		
To what extent do you think you have received sufficient information about what is considered permissible and impermissible use of GAI such as ChatGPT/Copilot in the physiotherapy programme?				
Very high degree High degree Neither nor Low degree Not at all	13 (10.6) 39 (31.7) 45 (36.6) 22 (17.9) 4 (3.3)	13 (11.6) 51 (45.5) 36 (32.1) 11 (9.8) 1 (0.9)	χ^2^ (4) = 7.57 *p* = 0.109	0.18
Have you read the study guide?				
No Yes	16 (13.0) 107 (87.0)	5 (4.5) 107 (95.5)	χ^2^ (1) = 4.26 *p* = 0.039	0.15
The text in the study guide clearly explains what is considered permissible and impermissible use of GAI such as ChatGPT/Copilot in the physiotherapy programme?				
Strongly agree Agree Neither nor Disagree Strongly disagree	14 (13.1) 46 (43.0) 35 (32.7) 10 (9.3) 2 (1.9)	15 (14.0) 52 (48.6) 32 (29.9) 6 (5.6) 2 (1.9)	χ^2^ (4) = 1.54 *p* = 0.820	0.08
How do you think your use of GAI such as ChatGPT/Copilot will change in the coming year?				
Slightly reduced Same as now Slightly increased Increased	1 (0.8) 55 (44.7) 52 (42.3) 15 (12.2)	5 (4.5) 60 (53.6) 32 (28.6) 15 (13.4)	Fisher’s Exact = 6.98 *p* = 0.070	0.17

Students also provided several suggestions for learning support related to their use of GAI. In total, 23 students in the 2024 survey and 12 students in the 2025 survey provided one or more suggestions. At the 2024 survey, the most frequent request concerned structured information, including teacher‑led introductions or lectures (*n* = 9), whereas none of the students requested this in the 2025 survey. Demonstration‑based learning activities, such as hands‑on sessions or workshops, were commonly suggested in both surveys. Requests for clarification of rules regarding the appropriate and ethical use of GAI were more frequent in the 2024 survey (*n* = 5) than in 2025 (*n* = 2). Smaller numbers of students expressed a need for prompting support, risk‑awareness training, or reported no need for support (Table 5).

**Table 5:** Suggested learning support activities for generative artificial intelligence (GAI) in the 2024 and 2025 surveys

Open-ended question “If you need a learning activity to support your use of GAI such as ChatGPT/Copilot, please write a suggestion for what such a learning-supporting activity could look like.”*			
**Category**	**Category description**	**Frequency** **2024 survey**	**Frequency** **2025 survey**
Prompting support	Help formulating effective prompts	2	3
Clarification of rules	Guidance on permitted use, ethics, and responsible use of GAI	5	2
Risk awareness	Instructions on AI limitations; errors, hallucinations, bias and how to critically evaluate outputs	1	2
Demonstration/practice	Hands-on sessions with concrete examples, practical tasks, scenarios, workshops, or opportunities to test GAI tools	6	4
Lecture/structured information	Request for a teacher-led introduction, lectures, structured overview covering basic functions, recommended practice, and tips for effective and responsible use of GAI	9	0
No need for support	Students who reported not needing any support with GAI	1	2
Total numbers of codes		24	13
*Frequencies represent coded responses; multiple responses were permitted. The question was answered by 23 students in the 2024 survey and 12 students in the 2025 survey			

### Generative AI literacy

GAILS-10 scores differed significantly between semester 1 students assessed before participation in the GAI learning activities and semester 3 students assessed after completion of the activities. Semester 3 students reported higher scores across all five GAILS-10 construct dimensions, with mean differences ranging from 0.5 to 1.4. The largest between-group difference was observed for prompt engineering ability, whereas the smallest difference was observed for ethical awareness. All differences were statistically significant (*p* < 0.01), with moderate to large between-group effect sizes (Cohen’s *d* = 0.4–1.2). Overall GAILS-10 scores were higher in semester 3 than in semester 1 (mean 5.8 vs. 4.9; total score 57.8 vs. 48.9: *p* < 0.001), corresponding to a large between-group effect size (*d* = 1.0) (Table 6).

**Table 6:** Students’ generative artificial intelligence literacy (GAILS-10) [32] in semester 1 and semester 3

GAIL Construct Dimension	Semester 1, *n* = 95, Mean (SD)	Semester 3, *n* = 80, Mean (SD)	Semester 3 – Semester 1, Mean Difference (95% CI), independent-samples t-test *p*-value	Effect size Cohen’s d (95% CI)
Basic Operational Skills	5.0 (1.2)	6.0 (0.8)	0.93 (0.64–1.23) *p* < 0.001	0.92 (0.61–1.23)
Prompt Engineering Ability	4.4 (1.3)	5.8 (0.8)	1.42 (1.09–1.74) *p* < 0.001	1.25 (0.92–1.57)
Quality Evaluation Ability	4.8 (1.2)	5.5 (0.8)	0.73 (0.42–1.04) *p* < 0.001	0.69 (0.39–1.0)
Innovative Application Ability	4.9 (1.2)	5.8 (0.9)	0.84 (0.52–1.16) *p* < 0.001	0.79 (0.48–1.10)
Ethical and Regulatory Awareness	5.3 (1.4)	5.8 (1.3)	0.55 (0.14–0.96) *p* < 0.01	0.40 (0.10–0.70)
GAILS-10, Mean (1–7)	4.9 (1.0)	5.8 (0.6)	0.89 (0.64–1.14) *p* < 0.001	1.03 (0.71–1.34)
GAILS-10 Sum (10–70)	48.9 (10.2)	57.8 (6.4)	8.93 (6.43–11.44) *p* < 0.001	1.03 (0.71–1.34)

*GAILS-10* 10-item Generative Artificial Intelligence Literacy Scale (higher scores indicating higher generative artificial intelligence literacy), *SD* Standard Deviation, *CI* Confidence Interval

## Discussion

The main findings of this study were threefold. First, physiotherapy students in the 2025 survey reported more frequent and diverse use of GAI for academic purposes than in the 2024 survey, with daily use reported by 57% compared with 13%. Second, a higher proportion of students in the 2025 survey perceived that GAI contributed to their learning. Third, semester 3 students assessed after completing the structured GAI learning activities reported higher GAI literacy across all five GAILS-10 dimensions than semester 1 students assessed before participation. Given the repeated cross-sectional design with non-equivalent, unlinked samples, these findings should be interpreted as differences between survey cohorts rather than individual change over time. Together, the findings indicate growing integration of GAI into students’ study practices and suggest that structured curricular adaptations and learning activities may help support GAI literacy in physiotherapy education. The higher proportion of daily GAI use reported in the 2025 survey aligns with another Swedish study, reporting that 64% of medical students used GAI chatbots in their studies [40]. Frequent GAI use has also been described at a psychology department in the UK, where most students and staff reported using GAI twice a week or more in the academic scenarios provided or while studying [41]. In contrast, a 2024 national survey of Italian physiotherapy students reported that 53.7% had never used chatbots for academic purposes [42], which suggests that adoption is shaped not only by tool availability but also by local educational cultures, guidance, and exposure. GAI has become increasingly accessible, likely contributing to the higher reported GAI use in the 2025 survey. The findings underscore the importance of AI literacy in healthcare education to ensure high-quality use in terms of ethics and critical thinking. A notable finding was that the respondents in the 2025 survey reported a broader range of GAI uses than those in the 2024 survey, particularly for brainstorming, question generation, and text refinement. This aligns with findings from the UK study, where both students and staff reported most commonly using GAI for idea generation (41%), explaining ideas (38%), and editing text (31%) [41]. Similarly, a systematic review found that health professions students primarily used GAI to clarify concepts, simulate scenarios, and generate practice materials, while GAI use for face-to-face group discussion and collaborative activities like teamwork was limited. This pattern suggests that GAI may promote a more individualised approach to learning [1], highlighting the need to design and implement additional learning activities that actively foster peer interaction and discussions. Students’ perceptions of GAI differed between the surveys. A higher proportion of respondents in the 2025 survey reported that GAI contributed to their learning to a “very high degree” compared with respondents in the 2024 survey (21.4% vs. 6.5%). Similar findings have been reported among Swedish medical students [40], and students in Communication Sciences and Disorders have also reported high levels of both usefulness and comfort when using GAI in their studies and clinical work [43]. In the present study, a higher proportion of students reported having read the study guide on GAI use in the 2025 survey and none requested structured teaching on GAI, compared to nine students in the 2024 survey. These findings are consistent with the implemented curriculum adaptations and GAI learning activities, which aimed to improve knowledge, clarify permitted use, and support critical and responsible GAI use. However, given the repeated cross-sectional design and unlinked samples, these patterns should not be interpreted as direct effects of the curricular changes or learning activities.

To our knowledge, this is one of the first studies to evaluate GAI learning activities in healthcare education using a structured GAI literacy scale. Only two small surveys have been conducted, and neither employed a validated GAI literacy questionnaire [44, 45]. In the current study, the overall GAILS-10 score provides a novel contribution by showing higher self-reported GAI literacy among semester 3 students assessed after the learning activities than among semester 1 students assessed before participation. This finding is relevant because GAI literacy is important for promoting appropriate GAI use in students’ learning [46], as for addressing security and ethical considerations in healthcare practice [47]. Further support for the importance of GAI literacy is provided by a study on nursing and midwifery students [19]. Their findings indicate that GAI literacy mediates self-directed learning and attitudes toward GAI among students pursuing careers in healthcare. However, a positive attitude alone does not promote self-directed learning. The findings emphasise the need for structured GAI education in healthcare curricula [19]. United Nations Educational, Scientific and Cultural Organisation (UNESCO) guidance for GAI in research and education emphasised the importance of GAI literacy to enable the use of such tools in fostering creativity, collaboration, critical thinking, and other higher-order thinking skills [48]. Furthermore, given the large variation in GAI attitudes and literacy levels, targeted GAI education and ethical considerations have been recommended for healthcare programmes [10, 49]. This, together with the ongoing reshaping of healthcare by GAI, which is already used for GAI-assisted diagnostics in 74% of European countries [50], emphasises the need for continued development of GAI learning activities and for measures of GAI literacy. Of the five dimensions in GAILS-10, prompt engineering ability showed the largest difference between semester 1 and semester 3 students. This finding is noteworthy, as prompting has been highlighted as a core competence in GAI use and as a skill that should be included in medical education [51]. One of the few studies on prompt engineering in healthcare education explored nursing students’ experiences of the use of GAI tools in developing care plans. The findings suggested that, with careful prompt refinement and critical evaluation, GAI could serve as a valuable supplement to, rather than a replacement for, humanistic nursing competencies [21]. The higher prompting ability reported by semester 3 students may reflect the focus of the GAI learning activities on practical use and prompt refinement. However, this should be seen alongside the increased societal adoption and everyday use of GAI, as previous research has demonstrated an exceptionally rapid global diffusion of GAI tools, particularly among younger and more highly educated users [52]. The observed higher GAI literacy scores suggest that GAI literacy is an emerging competence in physiotherapy education, extending beyond technical familiarity with GAI tools to include critical appraisal, ethical awareness, and responsible integration into professional reasoning. These competencies are increasingly relevant for future physiotherapy practice, where AI-supported tools may contribute to clinical decision-making, patient education, documentation, and evidence-informed practice. Curricula should therefore include structured learning activities and assessments that develop practical experience, critical judgement, and reflective use of GAI in relation to professional standards and ethics.

### Strengths and limitations

This study has several strengths. First, the intervention was implemented within an authentic course context and co-developed in collaboration with the medical library, making the findings relevant and applicable to real educational settings. This interdisciplinary collaboration may enhance the transferability of the learning activities beyond physiotherapy education while still ensuring relevance to the specific educational context of physiotherapy students. Second, coverage in the GAI literacy assessment was high, capturing nearly the entire cohort, which strengthens the study’s representativeness. Third, we reported both mean differences and standardised effect sizes with confidence intervals across five GAILS-10 dimensions, offering a nuanced picture of the magnitude and precision of observed between-group differences. An additional implementation-related strength was that the learning activities were embedded within mandatory sessions, which may have increased the visibility and perceived curricular importance of GAI. This suggests that visibility and curricular prioritisation may be relevant considerations when designing and implementing GAI-related education. A further strength was the use of the AMO framework, which guided the design of the learning activities and was also reflected in the assessment of GAI literacy. GAILS-10 was considered suitable because it is based on the AMO framework and retains the five-dimensional structure of the original GAIL scale, allowing assessment of specific components of GAI literacy aligned with the learning activities [32, 36]. However, the short form has not yet been independently validated, and the Swedish translated version used in this study was not culturally adapted or pilot-tested. In addition, the original scale was validated in the public sector rather than among higher education students.

Several limitations should be considered. The repeated cross-sectional design, based on unlinked cohorts and without a comparison group, precludes causal inferences regarding the effects of the curricular changes and learning activities. Because survey responses were anonymous, participants could not be matched across survey waves; thus, some students may have completed both surveys, and the independence of observations cannot be fully verified. However, anonymity was intentionally prioritised to encourage honest reporting of potentially sensitive GAI-related behaviours and perceptions. Consequently, the findings reflect differences between cohorts rather than individual change over time. The higher GAI literacy observed in 2025 may reflect not only the educational intervention but also cohort differences, prior exposure to GAI, and broader societal adoption of GAI during the study period. In addition, the semester distribution differed between survey waves, with fewer students from later semesters represented in 2025. As GAI use may increase with academic progression, this sampling difference should be considered when interpreting comparisons between cohorts. A limitation of this study is that the GAI use and perception questionnaire was purpose-developed by the authors and, although reviewed and refined by the research group, it was not formally pilot-tested or subjected to psychometric evaluation. Therefore, content validity, construct validity, and reliability were not formally established. All outcomes were self-reported, which introduces potential recall and social desirability biases. The dual role of the researchers as course instructors may have amplified such biases, although students completed the questionnaire anonymously. Particularly in cases where institutional policies prohibit the submission of AI-generated text for assessment, students may have under-reported actual GAI use, such as in generating new texts in assignments, which was uncommon both in 2024 and in 2025. The survey on GAI use and perceptions had a low response rate (51%–55%), which may reduce representativeness and introduce non-response bias for those findings. The single-institution setting may limit generalisability, although the findings are likely transferable to other courses within the Faculty of Medicine and Health Sciences. Moreover, the rapidly evolving nature of GAI tools means that literacy, use, and perceptions may shift over short intervals, potentially affecting the temporal stability of the results. Finally, the study focused on perceived literacy and did not include performance-based assessments or behavioural measures of GAI competence, which would strengthen construct validity and triangulate the self-reported outcomes.

## Conclusion

Physiotherapy students in the 2025 survey reported more frequent and diverse use of GAI and more positive perceptions of its use for learning than students in the 2024 survey. Semester 3 students assessed after participation in structured GAI learning activities reported higher GAI literacy than semester 1 students assessed before participation. These findings suggest a need for continued curricular adaptations, clear institutional guidance, and educational support to promote appropriate GAI use and strengthen students’ GAI literacy. Future longitudinal studies with linked individual-level data are needed to examine how GAI use and GAI literacy develop over time and to evaluate their associations with learning outcomes throughout professional education and clinical training.

## Supplementary Information


Supplementary Material 1.
Supplementary Material 2.


## Data Availability

The data generated and/or analysed during the current study are not publicly available as they contain information that could compromise the privacy of research participants. They are, however, available from the corresponding author upon reasonable request.

## Abbreviations

GAI: Generative Artificial Intelligence
ChatGPT: Chat Generative Pre-Trained Transformer
GAILS: Generative Artificial Intelligence Literacy Scale
GREET: Guideline for Reporting Evidence‑based Practice Educational Interventions and Teaching
LLMs: Large Language Models
PBL: Problem-Based Learning
UK: United Kingdom
UNESCO: United Nations Educational, Scientific and Cultural Organisation
