# Supplementary material for: Integrating generative AI into physiotherapy education: students’ use, perceptions, and generative AI literacy following curricular adaptations: a repeated cross-sectional programme evaluation
Source: BMC Med Educ. 2026 Aug 28;26:1399. doi: 10.1186/s12909-026-10256-0 (PMC13523361; doi:10.1186/s12909-026-10256-0)
Supplement: Supplementary file 1 [file 12909_2026_10256_MOESM1_ESM.docx]

Supplementary file 1. Alignment of Generative AI literacy learning activities with Ability-Motivation-Opportunity framework

| Semester and activity | Student Grouping and location | **Ability**  (Basic Operational Skills, Prompt Engineering Ability, and Quality Evaluation Ability) | **Motivation**  (Innovative Application Ability) | **Opportunity**  (Compliance and Ethical Awareness) |
| --- | --- | --- | --- | --- |
| **S1 – Introduction to GAI, part 1 (20 min)** Pre-recorded lecture on information retrieval including a 6-minute GAI introduction designed and delivered by a librarian. | Individually, learners’ home computer. | Introduces core GAI concepts: prompting, data security, bias, and ethical considerations. Builds foundational skills for using, understanding, and navigating GAI tools. | Reduces uncertainty, fosters confidence, and encourages willingness to apply GAI in academic work. | Explains university guidelines and provides a clear framework for safe and permitted use, and access to the recommended GAI tool. |
| **S1 –Introduction to GAI, part 2 (60 min)*** Information retrieval using multiple sources, including GAI, with case-based tasks and formative feedback, led by a librarian. | One-third of the class group** | Strengthens practical skills in searching and critically evaluating AI-generated content. | Enhances engagement and confidence in using GAI. | Provides access to university resources, supervision, and structured activities and feedback for responsible GAI use. |
| **S1 –** **Feedback on self-written text*** Through instructions in the study guide, students use GAI to obtain feedback on their own written text in two mandatory assignments, including a written reflection on how the feedback was used in one of the assignments. | Individually, learners’ home computer. | Develops skills in using GAI for formative feedback on writing, as well as the ability to critically evaluate and selectively apply AI-generated feedback. | Enhances perceived usefulness and self-regulated learning through reflection on AI-generated feedback on writing. | Provides a structured and legitimate opportunity to use GAI within the course guidelines. |
| **S2 – GAI-supported information retrieval (90 min)** Brief review of S1 and introduction to Scopus AI for database searching. Preparatory task tied to an assessment task involving the search and presentation of intervention study results, designed and delivered by a librarian. | Half-class group** | Builds database searching competence, including AI‑assisted term generation and article-suggestion functions. | Highlights GAI’s potential for efficient retrieval, motivating its use in academic information retrieval. | Provides clear usage guidelines and hands‑on experience with Scopus AI to support safe and regulated application. |
| **S3 – Critical review of AI-generated content. Part 1** Individual task using GAI to verify sources and factual claims against course literature. | Individually, learners’ home computer. | Builds technical and critical skills through prompting and source verification. | Strengthens confidence and willingness to use GAI critically. | Creates dedicated time and a structured setting for pedagogical engagement with GAI tools |
| **S3 – Critical review of AI-generated content. Part 2** Group assignment: synthesise results from part 1 and prepare a seminar presentation on how information was verified and which sources were used. | Quarter-class group, students’ choice of digital or in-person attendance. | Develops analytical and synthesis skills through collaborative review of AI‑generated scientific content. | Encourage peer collaboration and shared responsibility, increasing motivation for critical use. | Provides structured group work to collaboratively process and evaluate information, creating opportunities for responsible use of GAI. |
| **S3 – Critical review of AI generated content. Part 3 (90 min)*** Seminar presentation and ethical discussion led by physiotherapy educator and librarian. | Half-class group** | Enhances oral analysis, argumentation, and critical evaluation of AI-generated material. | Shared presentations and discussions increase engagement and social motivation to develop AI literacy. | Provides structured setting for experience exchange and expert facilitation for ethical reflection. |
| S = Semester, * = Mandatory, ** = In person at Medical Library | | | | |
