# Supplementary material for: Integrating generative AI into physiotherapy education: students’ use, perceptions, and generative AI literacy following curricular adaptations: a repeated cross-sectional programme evaluation
Source: BMC Med Educ. 2026 Aug 28;26:1399. doi: 10.1186/s12909-026-10256-0 (PMC13523361; doi:10.1186/s12909-026-10256-0)
Supplement: Supplementary file 2 [file 12909_2026_10256_MOESM2_ESM.docx]

**Supplementary File 2. Development and content of the generative AI use and perceptions questionnaire**

**1. Purpose**

The questionnaire explored physiotherapy students’ use and perceptions of generative AI (GAI) tools in their studies and included the following items:

- Frequency and purposes of GAI use
- Perceived contribution of GAI to learning
- Additional student-reported uses of GAI
- Clarity of guidance on permissible and impermissible use
- Need for learning support
- Suggested learning support activities
- Expected future use of GAI
- Trust in AI-generated responses (item added in the 2025 survey wave)

**2. Item generation**

The questionnaire items were developed by the research team (KS, YL, TE, SS) and informed by two main sources: previous research in physiotherapy education conducted within our research group and a review of the literature on students’ use and perceptions of GAI. The qualitative interview study by Lindbäck et al. (29) informed the development of items exploring physiotherapy students’ experiences, perceptions and anticipated use of GAI. A second study describing the structured development of learning and assessment tasks in response to GAI informed items addressing educational integration, permissible and impermissible use, AI literacy, learning support needs, and the prevention of GAI misuse in assessment contexts (30).

**3. Expert review**

The draft questionnaire was developed by KS, a researcher and senior physiotherapy educator, and subsequently reviewed by the research team including SS and YL, both researchers and senior physiotherapy educators, and TE, a physiotherapy educator with experience in GAI, pedagogy and digital learning. In addition, an educator from the university’s pedagogical development centre, with specific expertise in higher education pedagogy, contributed to the review process. Feedback from the reviewers was used to improve the clarity, relevance and comprehensiveness of the questionnaire items. Prior to survey administration, the programme director and all course leaders within the physiotherapy programme were invited to review and comment on the questionnaire. Before the 2025 survey wave, the questionnaire was further discussed among all members of the teaching staff in the physiotherapy programme, resulting in the inclusion of an additional item on students’ trust in AI-generated responses.

**4. Pilot testing**

No formal pilot testing of the questionnaire was conducted among students; instead, item refinement was undertaken through the review processes described above.

**5. Content validity**

Content validity was supported by previous research, expert review, educator discussions and student feedback from course evaluation meetings. Together, these sources helped ensure that the questionnaire addressed areas considered relevant to physiotherapy education and covered the intended domains.

**6. Measurement structure**

The questionnaire was intended to describe several separate areas of students’ GAI use and perceptions, rather than to generate a combined scale score. Therefore, individual items were analysed separately, and no overall questionnaire score was calculated.

----------------------------------------------------------------------------------------------------------------

**Questionnaire: Students’ use and perceptions of generative artificial intelligence (GAI)**

*The questionnaire was administered in Swedish. The version presented here has been translated into English using Microsoft Copilot.*

All analyses are conducted at group level. No individual person will be identifiable. By completing the questionnaire, you are considered to have consented to participate in the study.

**1) Which semester are you currently in?**

Options: Semester 1, 2, 3, 4, 5, 6

**2) How often do you use GAI such as ChatGPT/Copilot in your studies?**

Options: Every day, Once a week, Once a month, Never

**3) To what extent do you use GAI such as ChatGPT/Copilot to brainstorm ideas when studying?**

Options: Often, Quite often, Occasionally, Rarely, Never

**4) To what extent do you trust answers from GAI such as ChatGPT/Copilot?***

Options: Very high degree, High degree, Neither nor, Low degree, Not at all, Do not know

**5) To what extent do you use GAI such as ChatGPT/Copilot to write questions for yourself?**

Options: Often, Quite often, Occasionally, Rarely, Never

**6) To what extent do you use GAI such as ChatGPT/Copilot to rewrite/improve your written text to be submitted in an assignment?**

Options: Often, Quite often, Occasionally, Rarely, Never

**7) To what extent do you use GAI such as ChatGPT/Copilot to generate new text to be submitted as an assignment in your course?**

Options: Often, Quite often, Occasionally, Rarely, Never

**8) Are there any other ways you use GAI tools such as Copilot/ChatGPT in your studies, besides the previously mentioned purposes? If yes, please describe briefly.** (Open-ended question)

**9) To what extent do you think GAI such as ChatGPT/Copilot has contributed to your learning?**

Options: Very high degree, High degree, Neither nor, Low degree, Not at all

**10) Do you need a learning support element to be able to use GAI such as ChatGPT/Copilot optimally in your learning?**

Options: Yes, Uncertain, No

**11) Please suggest what such a learning support activity could look like:** (Open-ended question)

**12) To what extent do you think you have received sufficient information about what is considered permissible and impermissible use of GAI such as ChatGPT/Copilot in the physiotherapy programme?**

Options: Very high degree, High degree, Neither nor, Low degree, Not at all

**13) Do you think the text in the study guide clearly explains what is considered permissible and impermissible use of GAI such as ChatGPT/Copilot in the physiotherapy programme?**

Options: Strongly agree, Agree, Neither nor, Disagree, Strongly disagree, Have not read the study guide

**14) How do you think your use of GAI such as ChatGPT/Copilot will change in the coming year?**

Options: Slightly reduced, Same as now, Slightly increased, Increased

* Only used in the 2025 survey
